# Delayed hearing loss in congenital cytomegalovirus infection: systematic review and meta-analysis

**DOI:** 10.1016/j.bjorl.2026.101864

**Published:** 2026-07-23

**Authors:** Nicolau Moreira Abrahão, Arthur Menino Castilho, Eduardo Tanaka Massuda, Vagner Antonio Rodrigues da Silva

**Affiliations:** a Universidade Estadual de Campinas (UNICAMP), Department of Otolaryngology, Campinas, SP, Brazil; b Faculdade de Medicina de Ribeirão Preto da Universidade de São Paulo (FMRP), Ribeirão Preto, SP, Brazil

**Keywords:** Hearing loss, Late-onset hearing loss, Deafness, Cytomegalovirus, Neonatal screening

## Abstract

**Objectives:**

This study aims to evaluate the importance of universal screening for congenital Cytomegalovirus (CMV) infection in newborns using urine PCR to identify asymptomatic cases at birth, with or without early hearing impairment, and to monitor this population for the development of late-onset hearing loss.

**Methods:**

This systematic review followed the Preferred Reporting Items for Systematic Reviews and Meta-Analyses. The literature search was conducted through four online databases ‒ PUBMED, PUBMED PMC, BVS/BIREME, and EMBASE ‒ for papers published until January 2025. The Newcastle-Ottawa Scale was used to assess the risk of bias.

**Results:**

The search strategy identified 824 articles. Following the abstract screening, 22 studies were selected for full-text review, of which three met the criteria for data synthesis. All three studies were prospective, applied universal CMV screening through urine PCR, and followed patients with auditory brainstem response for at least four years. A meta-analysis was performed using a random-effects model with inverse-variance weighting and the Restricted Maximum Likelihood estimator. Among the three studies included in the assessment, 56,892 newborns were screened with urine samples. The meta-analysis found a proportion of prevalence of congenital CMV infection of 0.46%. The proportions of asymptomatic cases and late hearing impairment was 83.4% and 13.9%, respectively. Additionally, early hearing impairment was detected in 22 (12%) newborns, and one study demonstrated that 11% of asymptomatic patients developed long-term neurological sequelae in addition to hearing loss.

**Conclusion:**

Our meta-analysis evaluated epidemiological aspects of hearing loss associated with cCMV infection and found that a significant proportion of infants with asymptomatic infection develop late-onset hearing loss. The absence of structured screening risks missing these patients in long-term follow-up and may increase costs related to delayed diagnosis and investigation.

**Level of evidence:**

Level 2.

## Highlights

• The Worldwide rate of congenital cytomegalovirus infection ranges from 0.2% to 2%.
• About 10% of asymptomatic children will develop hearing impairment over the years.
• All those children won’t be identified because there isn’t a neonatal screening.
• Newborn screening enables auditory monitoring through Auditory Brainstem Response.

## Introduction

Cytomegalovirus (CMV) is a double-stranded DNA virus classified in the Herpesviridae family.^1^ Congenital Cytomegalovirus infection (cCMV) is the most common congenital viral infection, and it is the worldwide main cause of congenital hearing loss, excluding genetic causes,^2^, ^3^, ^4^, ^5^, ^6^ accounting for 6%–30% of pediatric hearing loss.^1^

The cCMV manifests symptoms in 10%–15% of patients, named as the symptomatic form. Symptoms include neonatal jaundice, petechial rash, respiratory distress, intra-uterine growth retardation, small for gestational age, hepatosplenomegaly, and microcephaly^3^^,^^7^^,^^8^ that often exhibit higher rates of mortality and an increased risk of long-term complications such as neurological sequelae, blindness, and deafness.^3^^,^^9^

Although approximately 85%–90% of all infected children are considered asymptomatic at birth, this group has a higher likelihood of developing neurological, visual, and hearing long-term sequelae.^3^^,^^7^ Moreover, approximately 22% of the so-called asymptomatic patients can present Sensorineural Hearing Loss (SNHL) at birth,^8^^,^^10^ which may progress over time.^4^^,^^9^

The mechanism of hearing loss related to CMV is not complete clarify. In the mouse model, CMV infection caused a sustained inflammatory response in the cochlea, with virus being found in perilymph and endolymph. Moreover, there is a signal of decreased density of Spiral Ganglion Neuron cells, the impairment of the homeostasis of stria vascularis, and a decrease in the connexin 43 expression. All these mechanisms led to the breakdown of the normal functions of the inner ear.^11^

Given that the rate of progression to sensorineural Late-Onset Hearing Loss (LOHL) is high in asymptomatic patients, and other late sequelae, such as neurological and visual impairment, can be present, the absence of follow-up among these children could delay interventions such as a cochlear implant and result in losses in language, visual, neurological, and motor development.^12^, ^13^, ^14^

Despite the high prevalence and long-term morbidity, a gold-standard method for screening has not yet been developed.^15^ Neonatal screening using urine is the most sensitive and specific method available,^16^ although saliva tests have improved in accuracy over recent years.^16^ Furthermore, saliva PCR may yield false-positive results because of CMV shedding in breast milk. When neonatal urine collection is difficult, saliva PCR may be used; however, positive results should be confirmed with urine PCR.^17^ Dried blood spot testing presents lower viral load in blood than in urine and saliva, reducing its sensitivity to levels between 28% and 75%.^18^^,^^19^ A recent meta-analysis evaluating the performance of DBS for cCMV across 15 studies reported a mean sensitivity of 62.3%.^20^

The current study assesses the importance of universal screening using urine PCR to determine the rate of cCMV infection, the prevalence of symptomatic and asymptomatic cases, and through a four-years of follow-up to identify the incidence of LOHL with asymptomatic cCMV infection.

## Methods

This current systematic review followed the Preferred Reporting Items for Systematic Reviews and Meta-Analyses (PRISMA). The protocol was registered in the International Prospective Register of Systematic Reviews (PROSPERO) on December 2, 2022.

The need for an ethical approval waiver was granted. The study was conducted between February 2022 and January 2025.

### Literature research and selection criteria

The literature research was conducted through four online databases ‒ PUBMED, PUBMED PMC, BVS/BIREME, and EMBASE ‒ for papers published until January 2025. There has not been a restriction on the language of publication. The research strategy keywords were: “Hearing Loss” OR “Deafness” AND “Cytomegalovirus” AND “Neonatal Screening. The same strategy was used in all online databases.

A librarian assisted in the planning and development of terms for the computerized search. After the search, the references from each database were exported to the Rayyan program QCRI (https://rayyan.qrci.org).

Once duplications were removed, two otolaryngologists independently assessed the articles for eligibility and defined the eligible literature. The third otolaryngologist resolved any divergences. The inclusion criteria were: (1) Prospective studies; (2) Articles with abstract and full text available; (3) Studies that assessed cCMV-infected newborns using urine samples collected up to two weeks of life; (4) Studies that assessed and followed patients using Auditory Brainstem Response (ABR); (5) Studies that followed patients for at least 48-months of life after discharge; (6) Studies published in an indexed journal; (7) All language studies.

The exclusion criteria were: (1) Retrospective studies; (2) Studies that exclusively involved newborns with higher risk factors for deafness or higher-risk pregnancies; (3) Screening studies through blood or saliva; (4) Studies using urine, blood and saliva screening tests that were analyzed separately; (5) Studies that follow patients up regarding on hearing less than 48-months; (6) Hearing follow-up conducted using methods other than ABR.

**Fig. 1:**
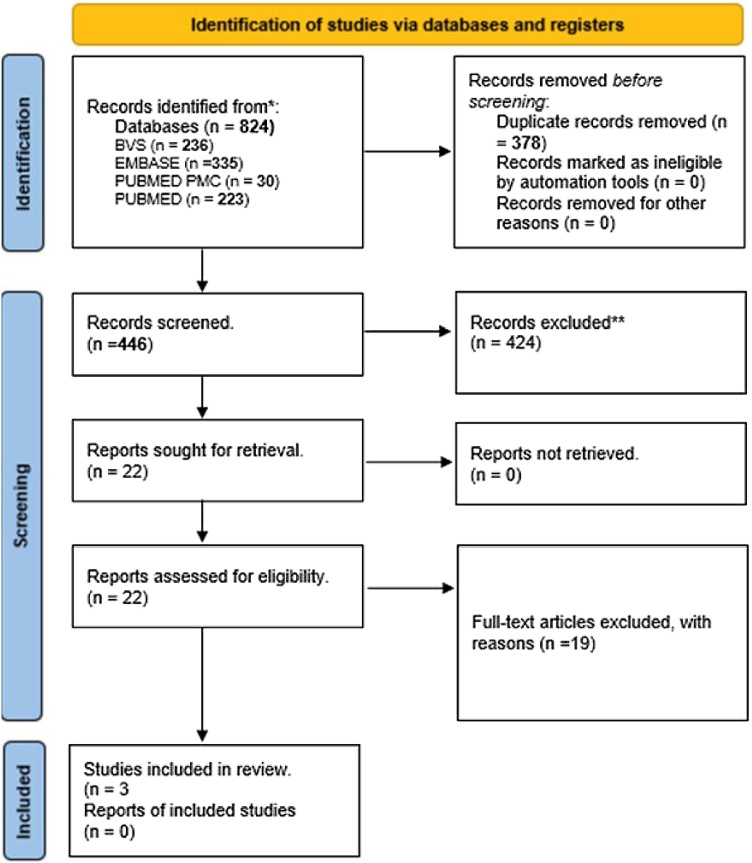
Flow diagram.

This full process is outlined in the PRISMA flowchart (Fig. 1).

### Quality assessment

The Newcastle-Ottawa scale (NOS; Score range: 1–9, with 9 indicating a lower risk of bias) was independently completed by two otolaryngologists. Studies with a score less than 6 were considered to have a high risk of bias. The scores obtained were 7 (Ahlfors et al.),^21^ 6 (Ina Foulon et al.),^22^ and 8 (Lanzieri et al.^9^ (Table 1).

**Table 1:** The Newcastle–Ottawa Scale.

First author	Selection				Comparability	Outcome			Total score
	Representativeness of exposed cohort	Selection of non-exposed cohort	Exposure ascertainment^a^	Outcome of interest not present at study start	Cohort comparability based on design or analysis^b^	Assessment	Adequate length of follow-up^c^	Adequate cohort follow-up^d^	
Ahlfors et al.	X	X	X	X	‒	X	X	X	7
Ina Foulon et al.	X	X	X	X	‒	X	X	‒	6
Lanzieri et al.	X	X	X	X	X	X	X	X	8

a Urine sample harvested at the first week.

b At least 48-months of follow-up.

c Less than 25% loss to follow-up.

d Compared the patient group with the control group.

### Data collection and synthesis

For each manuscript, the following data were collected: the study period, country, number of newborns tested, rate of cCMV infection, prevalence of symptomatic and asymptomatic cases, incidence of early and late-onset hearing loss, and incidence of other cCMV-related sequelae.

A narrative synthesis approach was used to summarize the findings from the selected studies. A table was created with study characteristics and findings.

### Statistical analysis

Statistical analyses were performed using R software, version 4.2.2. Proportion meta-analyses were conducted to estimate: (1) The prevalence of cCMV; (2) The proportion of asymptomatic cases among infected individuals, and (3) The proportion of LOHL among asymptomatic individuals. Quantitative syntheses were performed using a random-effects model, with inverse-variance weighting and the Restricted Maximum Likelihood estimator.

Given the limited number of included studies, proportion meta-analyses using a random-effects model were performed to describe between-study variability and provide pooled estimates with corresponding confidence intervals. The results of the quantitative syntheses are presented below, with graphical representation using forest plots.

## Results

The search strategy identified 824 articles in the database. After removing duplications, 446 articles remained. Following the data extraction, 22 studies were evaluated for full-text review. Of these, only three studies were included for data synthesis. The main reason for the exclusion was the absence of 48-months of follow-up (14 studies), followed by universal screening for cCMV (12); other test protocols like saliva or blood (9); monitoring during the follow-up with other tests than ABBR, and retrospective designs of studies. Five studies have only one reason to be excluded, eight have two reasons, followed by four studies that have three reasons, and two studies have five reasons. One study by Demmler-Harrison et al. met all inclusion criteria but was excluded because the authors did not clearly distinguish between CMV-infected cases and controls when assessing the progression of delayed-onset hearing loss. All this information is available in Table 2.

**Table 2:** Data synthesis detailed.

Author	Year	Newborns screened	Universal screening	Kind of study	Test protocol	Lenght of follow up (in months)	Follow up test	Included
Ahlfors K et al.	1984	10328	Yes	Perspective	Urine	48	ABR	Yes
Foulon I et al.	2008	14021	Yes	Prospective	Urine	48	ABR	Yes
Fuji T et al.	2017	575	No	Prospective	Urine	Not said	ABR	No
Iwasaki S et al.	2007	236	No	Prospective	Urine	48	ABR	No
Karimian P et al.	2016	1617	Yes	Prospective	Urine	12	ABR	No
Koyano S et al.	2016	23405	Yes	Prospective	Not said	24	ABR	No
Lanzieri T et al.	2017	32543	Yes	Prospective	Urine	> 48-months	ABR	Yes
Lorenzoni F et al.	2014	383	No	Prospective	Urine	Not said	ABR	No
Nagy A et al.	2004	70	No	Prospective	Urine	48	Not said	No
Yamada H et al.	2012	23405	Yes	Prospective	Not said	Not said	Not said	No
Yamada H et al.	2020	11736	Yes	Prospective	Urine	18	ABR	No
Yamaguchi A et al.	2016	23368	Yes	Prospective	Urine	18	ABR	No
Masarweh K et al.	2021	153	No	Prospective	Urine	24	ABR	No
Boppana S et al.	2005	96	No	Prospective	Saliva	34*	ABR	No
Foulon I et al.	2019	157	No	Prospective	Saliva or urine	> 48-months	ABR	No
Fowler K et al.	1999	388	No	Prospective	Saliva or urine	72	ABR	No
Inoue N et al.	2007	12	No	Retrospective	Blood and urine	Not said	Not said	No
Krishna S et al.	2020	444	No	Retrospective	Urine and Saliva	Not said	Not said	No
Stehel EK et al.	2006	483	No	Retrospective	Urine	Not said	ABR	No
Verbeeck J et al.	2008	526	No	Prospective	Urine - 1^st^ year	24	ABR	No
Yamamoto AY et al.	2019	11900	Yes	Prospective	Saliva	36*	ABR	No
Demmler-Harrison GJ et al.**	2020	32540	Yes	Prospective	Urine	> 48-months	ABR	No

### Studies characteristics

The study conducted by Ahlfors et al.^21^ between 1977 and 1982 identified 47 (0.5%) positive cases for cCMV in a sample of 10,328 newborns using urine collected in the first week. Of all positive cases, 9 (19%) were symptomatic while 38 (81%) were asymptomatic. All 47 patients were selected for follow-up, but only 43 completed it, of whom eight were symptomatic, and 35 were asymptomatic. None of them presented with early SNHL. None of them died. In the follow-up, 5 (11%) developed neurological sequelae and 3 (7%) developed LOHL, with two of them being asymptomatic at birth and one being symptomatic. Antiviral therapy was not administered at the time of diagnosis.

The study conducted by Ina Foulon et al.^22^ between1996 and 2006 tested 14,021 newborns using urine and found 74 (0.5%) positive cases for cCMV infection, with 4 (5%) symptomatic and 70 (95%) asymptomatic individuals. Of the 74 cases, 13 were lost to follow-up, and one died, leaving 60 patients remaining: three symptomatic and 57 asymptomatic. The minimum follow-up duration was 48-months.

Of these 60 patients, 13 presented with early SNHL: 12 from the asymptomatic group (21%), and one from the symptomatic group (33%). In these cases, sensorineural hearing impairment was unilateral in five cases and bilateral in eight cases, ranging from mild (two cases), moderate (three cases), severe (five cases), and profound (two cases). Three other patients (5%) developed LOHL during the follow-up. The authors did not specify whether they were from the symptomatic or asymptomatic group. Antiviral therapy was not done at the time of diagnosis.

The study conducted by Lanzieri et al.^9^ between 1982 and 1992 screened 32,543 newborns using urine samples and identified 135 (0.4%) cCMV infection cases. Of these, 92 asymptomatic patients (68%) were included in a prospective study, which compared them with a control group of 51 non-infected newborns. Newborn sex, pregnancy duration, maternal age, and multiparity did not differ statistically between groups (p > 0.05). Nine (10%) patients with early SNHL were identified.

Both groups were followed using ABR for 18-years. Six out of 92 cases missed follow-up, leaving 86 remaining cases. The authors demonstrated that the prevalence of children with NSHL increased from 7% at 3-months of life to 14% at 5-years of age and further to 25% at 18-years of age in the cCMV group. Conversely, among the control group, the prevalence of NSHL varied from 0% at 5-years of age to 8% at 18-years of age (HR = 4.0; 95% Confidence Interval: 1.2–14.5; p = 0.02). The risk of SNHL development was three times greater among cCMV patients than controls (HR = 3.0; 95% CI 0.9–10.5; p = 0.08), although not statistically significant.

Of the 9 (10%) patients with early SNHL, eight initially had unilateral loss. Over the years, 75% of them developed SNHL in the contralateral ear. On the other hand, 11 patients (14%) developed LOHL.

Still in the same study, the severity of SNHL increased over the years. In the last assessment, 12 (60%) of the 20 SNHL cases had moderate to severe impairment in the worst ear. The authors estimated that the proportion of patients who will need a hearing device increased from 10% at 12-months of age to 14% at 18-years of age. Similarly, the proportion of patients who will need a cochlear implant increased from 1% at 25-months of age to 2% at 5-years of age.

Among the three studies included for quantitative analysis, 56,892 newborns were screened for cCMV using urine samples. Of these, 256 (0.4%) tested positive for the congenital infection, of whom 56 (21%) were symptomatic and 200 (79%) asymptomatic. Twenty-two asymptomatic cases missed the follow-up, leaving 178 patients who were followed for at least four years. Early SNHL was detected in 22 (12%) newborns, with at least 12 cases from the asymptomatic group. LOHL was detected in 14 (7.5%) newborns.

### Meta-analysis

**Fig. 2:**
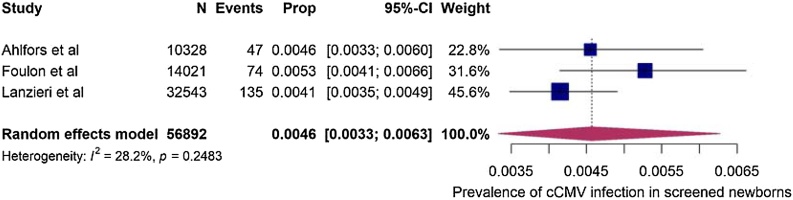
Prevalence of cCMV infection in screened newborns: random-effects meta-analysis.

The meta-analysis of cCMV infection prevalence included the three studies. Individual prevalence’s ranged from 0.41% to 0.53%, corresponding to 0.46% (95% CI 0.33%–0.60%) in Ahlfors et al.,^21^ 0.53% (95% CI 0.41%–0.66%) in Foulon et al.,^22^ and 0.41% (95% CI 0.35%–0.49%) in Lanzieri et al.,^9^ with relative weights in the random-effects model of 22.8%, 31.6%, and 45.6%, respectively. The pooled estimate from the random-effects model was 0.46% (95% CI 0.33%–0.63%). Between-study heterogeneity (I^2^) was 28.2% and not statistically significant (p = 0.248). The graphical representation of this analysis is shown in Fig. 2.

**Fig. 3:**
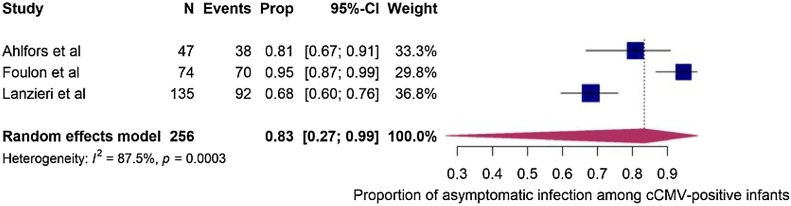
Proportion of asymptomatic infection among cCMV-positive infants: random-effects meta-analysis.

The meta-analysis of the proportion of asymptomatic cases among newborns infected with cCMV included the three studies. Individual proportions ranged from 68.2% to 94.6%, corresponding to 80.9% (95% CI 66.7%–90.9%) in Ahlfors et al.,^21^ 94.6% (95% CI 86.7%–98.5%) in Foulon et al.,^22^ and 68.2% (95% CI 59.6%–75.9%) in Lanzieri et al.,^9^ with relative weights in the random-effects model of 33.3%, 29.8%, and 36.8%, respectively. The pooled estimate from the random-effects model was 83.4% (95% CI 26.7%–98.6%). Substantial heterogeneity was observed across studies (I^2^ = 87.5%; p = 0.0003). The graphical representation of this analysis is shown in Fig. 3.

The proportion of LOHL among newborns with asymptomatic cCMV at birth included two studies, totaling 121 asymptomatic individuals followed and 23 events of LOHL. Ahlfors et al.^21^ reported a proportion of 5.7% (95% CI 0.7%–19.2%), whereas Lanzieri et al.^9^ reported a proportion of 24.4% (95% CI 15.8%–34.9%), with relative weights of 41.7% and 58.3% in the random-effects model, respectively. The pooled estimate from the random-effects model was 13.9% (95% CI 0.0%–99.98%). Foulon et al.^22^ were not included in this analysis because they did not report stratification of LOHL cases, precluding specific extraction of events among asymptomatic individuals.

Given the small number of included studies, the wide imprecision of the pooled confidence interval, and the observed heterogeneity, this analysis should be interpreted as essentially descriptive, with the primary aim of providing a comparative presentation of the individual study results.

**Fig. 4:**
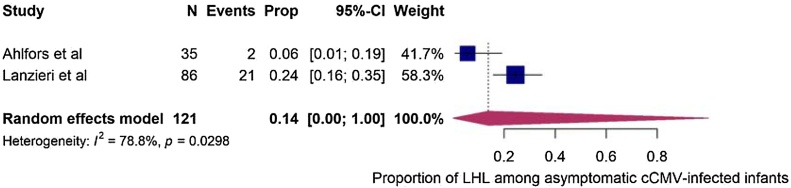
Proportion of late-onset hearing loss among asymptomatic cCMV-infected infants: descriptive random-effects meta-analysis.

The graphical representation of this analysis is shown in Fig. 4.

## Discussion

### Comparison across the studies

All three studies were conducted at single hospitals located in Sweden,^21^ Belgium,^22^ and the United States.^9^ This suggests a biased sample, as all studies were conducted in Western developed countries, which may help explain these prevalence estimates, as they fall at the lower end of the range reported in the literature.^2^, ^3^, ^4^, ^5^, ^6^^,^^23^ Additionally, it is well known that CMV seroprevalence in women of childbearing age is higher in developing countries.^24^^,^^25^ The largest cohort consisted of 32,543,^9^ followed by 14,021,^22^ with the smallest comprising 10,328.^21^ The earliest research was carried out between 1977 and 1982,^21^ followed by the study from 1982 to 1992,^9^ and the most recent one occurred from 1996 to 2006.^22^ No recent studies were identified by the authors that met all the inclusion and exclusion criteria.

The rate of asymptomatic cases ranged from 68% to 95%, and the heterogeneity observed among studies was high. The explanation for this variation is unclear, as the criteria used to define symptomatic cases were the same across studies, based on symptoms at birth, including neonatal jaundice, petechial rash, respiratory distress, intra-uterine growth retardation, small for gestational age, hepatosplenomegaly, and microcephaly. Although these Western countries share relative cultural, socioeconomic, and historical homogeneity, comparing data across nations can be complex.

### Comparison across studies at follow-up

Ahlfors et al.^21^ monitored children for general development, as well as neurological, ophthalmological, and hearing status from three months of age to four years. No patients manifested ophthalmological impairment. Five patients (11%) exhibited neurological sequelae, with two symptomatic (25%) and three asymptomatic (9%). LOHL was found in three patients (7%), representing 5.7% of the meta-analysis sample. This highlights the importance of monitoring these patients, not only to diagnose LOHL but also to identify associated neurological sequelae,^8^^,^^26^ which is in line with prospective studies reporting a cumulative incidence of neurodevelopmental impairment ranging from 0% to 9.1% in asymptomatic cCMV-infected children followed for one to six years.^17^^,^^27^^,^^28^

The other two studies^9^^,^^22^ focused solely on monitoring children for hearing development, one for a period of five years^22^ and the other for 18-years.^9^ In the study by Foulon et al.,^22^ 3 (7%) patients were diagnosed with LOHL at 8-, 15-, and 79-months of age. This finding contrasts with additional literature, which demonstrated that up to 37.5% of asymptomatic children developed late-onset hearing loss with a median of 44-months.^29^ Of the three children, two of them exhibited unilateral hearing loss – one with severe hearing loss and the other with moderate loss. The infant with bilateral LOHL had a severe loss in one ear and moderate loss in the other. An additional five patients (11%) exhibited progressive hearing loss. The incidence of hearing loss in asymptomatic infants was found to be 21%, while previous studies have put the incidence of SNHL at 6% to 25.^29^^,^^30^ The potentially progressive nature of early cCMV-related SNHL has also been suggested by studies not included in the analysis, which emphasizes the importance of long-term audiological monitoring in children diagnosed with congenital CMV infection.^31^

Lanzieri et al.^9^ followed two groups of patients for 18-years. The first group consisted of asymptomatic cCMV children and was compared to a group of non-infected patients. This study demonstrated how late hearing loss can occur, as SNHL increased from 7% at 3-months of age to 14% at 5-years, and further to 25% at 18-years of age in the cCMV group, presenting a proportion of 24.4% in the meta-analysis sample.

Conversely, the study demonstrated that the greatest differences between the two groups occurred in the first five years. While the prevalence of SNHL doubled among cCMV patients, it remained at 0% in the control group. From ages 6–18 years, however, the prevalence was almost similar between case patients and controls: 11% and 8%, respectively. Eleven patients in this study presented LOHL. Eight were from the asymptomatic cases group, and 3 were from the non-infected group. This extended follow-up period contrasts with most studies in the literature, which typically followed infected patients only until the age of four. Further studies with longer follow-up will be important for guiding the ideal duration of audiological monitoring for hearing loss related to cCMV. Some studies have suggested extending hearing follow-up until the age of six years of age.^3^^,^^32^

Additionally, the study highlights that asymptomatic children with early impairment in one ear are at greater risk of subsequent delayed SNHL in the normal hearing ear compared with the controls. Six patients initially presenting early SNHL in one ear, developing contralateral SNHL over the years.

### Pooled data analysis

Universal screening for cCMV was performed in 56,892 newborns across all three studies, thereby minimizing the risk of selection bias, as all individuals within the target population were screened, regardless of risk factors or clinical presentation. Urine samples were collected within the first week of life in two studies^21^^,^^22^ and within the first three days^9^ of life in one study. This reinforces the idea that if collected after 2–3 weeks of life, it is not possible to exclude postnatal CMV infection.^33^ The method for urine collection, as well as the analysis method and timing, were consistent across the studies.

The pooled prevalence of cCMV infection was 0.46%, which falls at the lower bound of the prevalence range described in the literature.^2^, ^3^, ^4^, ^5^, ^6^^,^^23^ The 83% proportion of asymptomatic cases is supported by the literature.^26^^,^^34^^,^^35^

Early hearing impairment was found in 22 (12%) newborns of the pooled 199, who tested positive for cCMV. Of these, 12 newborns (55%) were asymptomatic at the study by Ina Foulon.^22^ The study by Lanzieri et al.^9^ did not distinguish between symptomatic and asymptomatic cases of early hearing impairment. Finally, Ahlfors et al.^21^ found no cases of early hearing impairment.^21^

The proportion of LOHL in the meta-analysis sample was 13.9%. The prevalence of LOHL related to cCMV in asymptomatic patients is 22% according to the literature.^8^^,^^10^

### Impact of cCMV infection

Since LOHL related to cCMV typically manifests after three months of age, most asymptomatic newborns will pass neonatal hearing screening (otoacoustic emissions or ABR), as established and recommended by the Joint Committee on Infant Hearing, and will not be identified as being at increased risk for hearing loss.^34^^,^^36^ Hicks et al. demonstrated that only 14% of children with SNHL related to cCMV were identified through newborn hearing screening.^37^ As a result, undetected cases that go unmonitored have delayed diagnosis of late-onset hearing loss, missing the critical intervention window by the ninth month of age, during which therapeutic outcomes are more favorable for language development.^35^^,^^38^

Newborn screening for cCMV enables auditory monitoring through ABR tests and early intervention that can potentially reduce the necessity for additional tests for genetic causes^39^, ^40^, ^41^, ^42^, ^43^, ^44^ and embryologic malformation, like Enlarged Aqueduct Vestibule, related to SNHL.^45^ A shortening of the diagnosis through targeted neonatal screening might reduce the direct costs of the etiological diagnosis of deafness, considering the expenses associated with genetic and imaging tests.^33^^,^^46^ It also might help in monitoring other potential sequelae over time, such as cognitive and motor deficits. Estimates suggest that 18% of asymptomatic patients will develop one or more sequelae over time.^26^ Among the three articles in this synthesis, Alfhors et al.^21^ demonstrated that 11% of asymptomatic patients developed long-term neurological sequelae in addition to hearing loss.

Reviewing data published by the USA has demonstrated that up to the age of four, early and late sensorineural hearing impairment related to asymptomatic cases of cCMV account for 18% of all causes of deafness at this age.^39^ This estimate is likely underestimated due to the absence of routine hearing screening in developed countries.^47^ Other studies demonstrated that the likelihood of delayed hearing impairment is greater in CMV-positive children compared to non-infected children, even after 24-months of follow-up.^48^

In developing countries, these numbers can be even higher, as the prevalence of CMV infection among women of childbearing age is greater.^49^^,^^50^

### Limitations of the study

This study has several limitations. The three studies analyzed were conducted in developed Western countries, which may introduce selection bias. Furthermore, the small number of studies reduces the statistical power of our meta-analysis, limits generalizability, and highlights the need for robust clinical research. Although restricting inclusion to studies using urine PCR substantially reduced the number of eligible studies, this approach facilitated comparability across studies by minimizing an additional source of methodological variability. Nevertheless, future research should also evaluate the role of saliva PCR in this context.

Another limitation is the fact that all three studies did not treat the asymptomatic cases and did not specify if the symptomatic cases were treated with antiviral therapy. The antiviral therapy has been recommended only for infants with moderate/severe symptoms.^17^^,^^51^ Although current evidence suggests potential therapeutic benefits of antiviral treatment for children with isolated or delayed-onset SNHL, treatment is not recommended for asymptomatic/mild cases and/or isolated hearing loss at birth^17^ due to the risk of neutropenia.^51^

## Conclusions

Our meta-analysis evaluated epidemiological aspects of sensorineural hearing loss associated with cCMV infection and found an infection rate of 0.46%, with 83.4% of cases asymptomatic at birth. Notably, 13.9% of infants with asymptomatic infection develop LOHL, supporting the notion that cCMV remains a leading non-genetic cause of hearing loss. The absence of structured screening risks missing these patients in long-term follow-up and increasing costs related to delayed diagnosis and investigation.

## ORCID ID

Nicolau Moreira Abrahão: 0000-0002-0215-0948

Arthur Menino Castilho: 0000-0002-9024-8004

## Funding

None.

## Data availability statement

The authors declare that all data are available in repository.

## Declaration of competing interest

The authors declare no have conflict of interest.
